# Amelioration of LPS-Induced Inflammation Response in Microglia by AMPK Activation

**DOI:** 10.1155/2014/692061

**Published:** 2014-06-17

**Authors:** Chin-Chen Chen, Jiun-Tsai Lin, Yi-Fang Cheng, Cheng-Yi Kuo, Chun-Fang Huang, Shao-Hsuan Kao, Yao-Jen Liang, Ching-Yi Cheng, Han-Min Chen

**Affiliations:** ^1^Institute of Applied Science and Engineering, Catholic Fu-Jen University, New Taipei City 24205, Taiwan; ^2^Energenesis Biomedical Co., Ltd., New Taipei City 24205, Taiwan; ^3^Institute of Biochemistry and Biotechnology, Chung Shan Medical University, Taichung City 40201, Taiwan; ^4^Department of Life Science, Catholic Fu-Jen University, New Taipei City 24205, Taiwan; ^5^Research Center for Industry of Human Ecology, Graduate Institute of Health Industry Technology, Department of Cosmetic Science, Chang Gung University of Science and Technology, Taoyuan 33303, Taiwan

## Abstract

Adenosine 5′-monophosphate-activated protein kinase (AMPK) is a key regulator of cellular energy homeostasis via modulating metabolism of glucose, lipid, and protein. In addition to energy modulation, AMPK has been demonstrated to associate with several important cellular events including inflammation. The results showed that ENERGI-F704 identified from bamboo shoot extract was nontoxic in concentrations up to 80 *μ*M and dose-dependently induced phosphorylation of AMPK (Thr-172) in microglia BV2 cells. Our findings also showed that the treatment of BV2 with ENERGI-F704 ameliorated the LPS-induced elevation of IL-6 and TNF-*α* production. In addition, ENERGI-F704 reduced increased production of nitric oxide (NO) and prostaglandin E2 (PGE2) via downregulating the expression of inducible nitric oxide synthase (iNOS) and cyclooxygenase 2 (COX-2), respectively. Moreover, ENERGI-F704 decreased activated nuclear translocation and protein level of NF-*κ*B. Inhibition of AMPK with compound C restored decreased NF-*κ*B translocation by ENERGI-F704. In conclusion, ENERGI-F704 exerts inhibitory activity on LPS-induced inflammation through manipulating AMPK signaling and exhibits a potential therapeutic agent for neuroinflammatory disease.

## 1. Introduction

Homeostasis of proinflammatory and anti-inflammatory response in brain is shifted towards a proinflammatory state with age, so neuroinflammation has been implicated as an important etiological factor in several aging-related neurodegenerative diseases, such as Alzheimer's disease and Parkinson's disease [1–3]. Due to the involvement of inflammation in pathogenesis, many studies were devoted to the therapy of neurodegenerative diseases using anti-inflammatory strategies [4]. Microglia are myeloid-lineage cells residing in the central nervous system. As a neuron protector, microglia are sensitive to microenvironment and readily become activated in response to immunological stimuli, toxin, or injury [5]. Upon activation, however, microglia secrete a variety of proinflammatory cytokines or other cytotoxic factors, which are believed to exacerbate neurodegeneration. It has been reported that lipopolysaccharides (LPS) and/or interferon-*γ* enhanced the production of NO in microglia via inducible nitric oxide synthase (iNOS) and caused neuron death within 48 hours [6]. In addition, the increased proinflammatory cytokines such as TNF-*α*, IL-1*β*, and IL-6 in either cell culture or animal models induce neuron degeneration [7–12].

NF-*κ*B, a transcriptional factor, regulates several proinflammatory cytokines and inflammation-related protein expression such as TNF-*α*, IL-1*β*, IL-6, COX-2, and iNOS [13]. Upon stimulation, activated I*κ*B kinase (IKK) phosphorylates I*κ*B, which results in the dissociation of NF-*κ*B-I*κ*B complex and thereby translocation of active NF-*κ*B into nucleus. Activation of NF-*κ*B has been found in several neurodegenerative diseases including Alzheimer's disease, Parkinson's disease, and Huntington's disease [14–16] and has also been considered as an important target for therapy of neurodegenerative diseases.

AMP-activated kinase (AMPK) is a key regulator of energy homeostasis and metabolic stress [17]. Conditions of glucose deprivation, ischemia, or oxidative stress activate AMPK through upstream kinases such as liver kinase B1 (LKB1) and Ca^2+^/calmodulin dependent kinase kinase *β* (CaMKK*β*) by phosphorylation on conserved Thr-172 residue of *α* subunit [18, 19]. Upon activation, AMPK directly phosphorylated downstream targets such as acetyl-CoA carboxylase to inhibit anabolic pathways such as fatty acid/cholesterol synthesis, protein synthesis, and gluconeogenesis [20] and increase cellular ATP level. In particular, recent studies reveal that AMPK might also be involved in modulating inflammatory response [21–24]. It has been demonstrated that an AMPK activator, 5-aminoimidazole-4-carboxamide ribose (AICAR), suppressed LPS-induced proinflammatory secretion and attenuated nuclear factor-*κ*B (NF-*κ*B) activation in glial cells [25].

In the present study, we investigated the anti-inflammatory effects of ENERGI-F704, a purine compound identified from bamboo (*Phyllostachys edulis*) shoot extract [26], in microglia BV2 cells stimulated by LPS. We examined the effects of ENERGI-F704 on AMPK activation. We also assessed its anti-inflammation functions by monitoring the secretion of proinflammatory cytokines, the production of NO and PGE2, and the expression of corresponding enzymes in various conditions. It was found that ENERGI-F704 might attenuate LPS-induced inflammation and the nuclear translocation of NF-*κ*B via AMPK activation in BV2 cells.

## 2. Materials and Methods

### 2.1. Reagents

All reagents were purchased from Sigma-Aldrich (St. Louis, MO, USA) except where otherwise specified. Dulbecco's modified Eagle's medium (DMEM) and fetal bovine serum (FBS) were purchased from Invitrogen (Carlsbad, CA, USA). ENERGI-F704 was a proprietary compound generously provided by Energenesis Biomedical Co., Ltd. (New Taipei, Taiwan).

### 2.2. Cell Culture

The murine microglial cell line (BV2) was given by Professor Kao (Chung Shan Medical University, Taichung, Taiwan) and routinely maintained in Dulbecco's modified Eagle's medium (DMEM) supplemented with 10% fetal bovine serum (FBS), 4 mM L-glutamine, 2 mM sodium pyruvate, and 100 *μ*g/mL penicillin-streptomycin (Invitrogen GibcoBRL, Carlsbad, CA, USA). Cells were incubated at 37°C under 5% CO_2_ and 95% relative humidity. The cells used in this experiment were between passages 3 and 8.

### 2.3. *In Vitro* Cytotoxicity Assay

The cytotoxicity of ENERGI-F704 in BV2 cells was analyzed by XTT assay. In Brief, BV2 cells were seeded into 96-well microplates in a density of 1 × 10^4^ cells/well. After incubation overnight, cells were treated with various concentration of ENERGI-F704 (0, 5, 10, 20, 40, or 80 *μ*M) for 24 h. After the end of treatment, 125 *μ*L of XTT (2,3-bis-(2-methoxy-4-nitro-5-sulfophenyl)-2H-tetrazolium-5-carboxanilide) reagent was added to the final concentration of 1 mg/mL. Then, the plates were incubated at 37°C for further 4 h in the dark. The absorbance was measured at 490 nm with a reference wavelength set at 690 nm using VersaMax ELISA microplate reader (Molecular device, Sunnyvale, CA, USA). Data was presented as relative absorbance values to untreated cell.

### 2.4. Western Blot Assays

Cells were collected, washed twice with ice-cold PBS (PH 7.4), and lysed in cell lysis buffer containing 10 mM Tris-HCl pH 7.5, 150 mM NaCl, 1 mM EDTA, 0.5% Triton-X 100, 1x protease inhibitor cocktail (Roche, Basel, Switzerland), and 1x PhosSTOP phosphatase inhibitor cocktail (Roche) at 4°C for 30 min. Cell lysate was centrifuged at 15,000 g, 4°C for 1 min, and the supernatant was stored at −70°C until further analysis. 30 mg of protein samples was subjected to 10% SDS-PAGE and subsequently transferred onto Immobilon polyvinylidene difluoride (PVDF) membranes (Millipore, Bedford, MA, USA). After blocking with 5% BSA in PBS, the membranes were incubated with primary antibodies, including anti-phospho-AMPK (Thr172) antibody (1 : 2000, number 2535, Cell Signaling Technology, Danvers, MA, USA), anti-AMPK*α*1 antibody (1 : 2000, number 2603, Cell Signaling Technology), anti-iNOS antibody (1 : 2000, number 2977, Cell Signaling Technology), anti-COX-2 antibody (1 : 2000, number 12282, Cell Signaling Technology), anti-p65 antibody (1 : 2000, number 8242, Cell Signaling Technology), or anti-actin antibody (1 : 5000, NB600-501, Novus Biologicals, Littleton, CO, USA) at 4°C overnight. Resulting membranes were washed and incubated with corresponding secondary antibodies coupled with horseradish peroxidase in 1 : 20000 dilution at room temperature for 1 h. Chemiluminescence of the immunoreactive bands was developed by LumiFlash Prime Chemiluminescent Substrate, HRP (Visual Protein, Taipei, TW), and detected by Kodak XAR-5 film (Rochester, NY, USA). The images were scanned and quantified using ImageJ software (http://imagej.nih.gov/ij/).

### 2.5. Enzyme-Linked Immunosorbent Assay (ELISA)

Production of proinflammatory cytokine and PGE2 was analyzed using ELISA assay. The supernatant of cell culture was harvested and assessed. The cytokines IL6 and TNF-*α* were evaluated using Mouse DuoSet ELISA kits (R&D Systems, Minneapolis, MN, USA) and the extracellular PGE2 was assessed using Prostaglandin E2 parameter assay kit (R&D Systems). All the manipulations were performed following the manufacturer's protocol.

### 2.6. Nitric Oxide Determination

Production of nitric oxide was measured using the Griess assay. In brief, 100 *μ*L of supernatant sample was mixed with equal volume of Griess reagent containing 1% sulfanilamide, 0.1% N-(1-naphthyl)-ethylenediamine dihydrochloride, and 5% H_3_PO_4_. After 5 min incubation, the absorbance was measured at 550 nm with a reference wavelength set at 630 nm using VersaMax ELISA microplate reader. A standard curve made from a series of standard nitrite concentrations (0, 6.25, 12.5, 50, 66.7, 100, and 200 *μ*M) was used for sample calibration.

### 2.7. NAD Assay

NAD level was determined by NADH cycling enzymatic reaction using NAD/NADH quantification colorimetric kit (Biovision, Milpitas, CA, USA) according to the manufacturer's instructions. In brief, 2 × 10^5^ cells were harvested and extracted with 400 *μ*L NAD/NADH Extraction Buffer by two freeze/thaw cycles (20 min on dry-ice, then 10 min at RT), followed by vortex and centrifugation at 14000 rpm for 5 min. The supernatants were examined for the total level of NAD plus NADH by mixing with NAD Cycling Mix for conversion of NAD to NADH and measured for the free NADH by heat decomposing of NAD at 60°C for 30 min. The colorimetric assay of NADH was developed by adding NADH developer and it was incubated at RT for 1 h. The absorbance was measured at 450 nm using VersaMax ELISA microplate reader (Molecular device, Sunnyvale, CA, USA). Data was presented as relative absorbance values to untreated cell. A standard curve made from a series of standard NADH amounts (0, 20, 40, 60, 80, and 100 pmol) was used for sample calibration. The NAD level was calculated as NAD = total of NAD + NADH − NADH. The data were normalized with total cell lysate protein (NAD/mg protein) and presented as relative concentration to untreated cell.

### 2.8. Immunocytochemistry

Cells were cultured on glass coverslips in the DMEM based medium. The cultures were treated with LPS incorporating with ENERGI-F704 or compound C in indicated concentrations for 1 h at 37°C. The cells were fixed with 4% paraformaldehyde and subsequently incubated with anti-p65 antibody (1 : 200, number 8242, cell Signaling Technology) at 4°C overnight followed by the incubation with Alex Fluor 488-labeled secondary antibody (1 : 1000, Abcam, Cambridge, UK) for 1 h at room temperature. The nuclei of cells were counterstained with DAPI. Coverslips were mounted with ibidi mounting medium (ibidi GmbH, Martinsried, Germany) and the immunofluorescence was imaged by OLYMUS 1X71 inverted microscope. The resulting images were quantified using ImageJ software.

### 2.9. Statistical Analysis

All results were presented as means ± SEM of three independent experiments. Statistical analysis was performed by one-way ANOVA or two-way ANOVA using SPSS software (Armonk, NY, USA).

## 3. Results

### 3.1. ENERGI-F704 Activates AMPK in BV2 Cells

**Figure 1:**
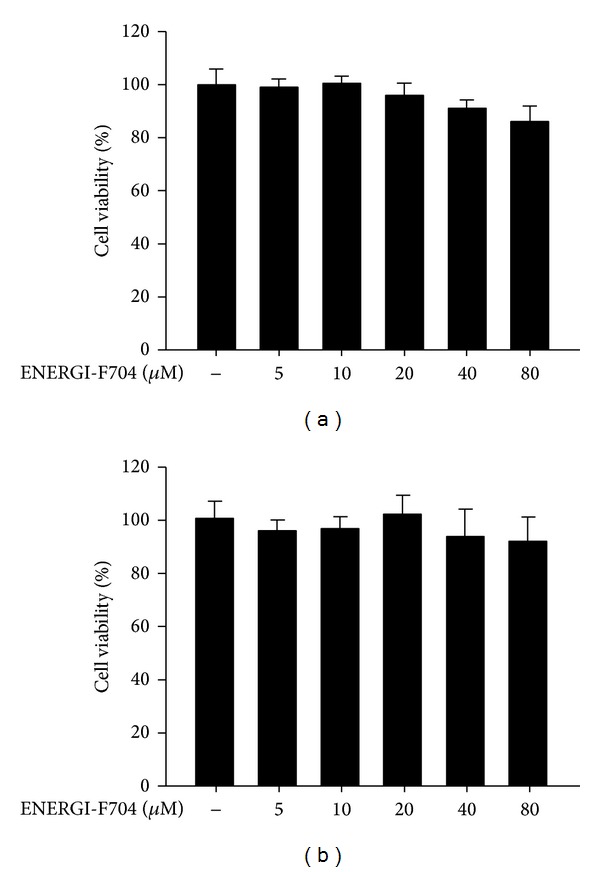
Cytotoxicity of ENERGI-F704. BV2 cells induced either without (a) or with (b) LPS were treated with indicated concentrations of ENERGI-F704 for 24 h. The cell viability of each condition was analyzed using XTT assay. The relative cytotoxicity is calculated as relative absorbance values to untreated cell. Data are statistically analyzed by one-way ANOVA and presented as the mean ± SEM of three independent experiments.

**Figure 2:**
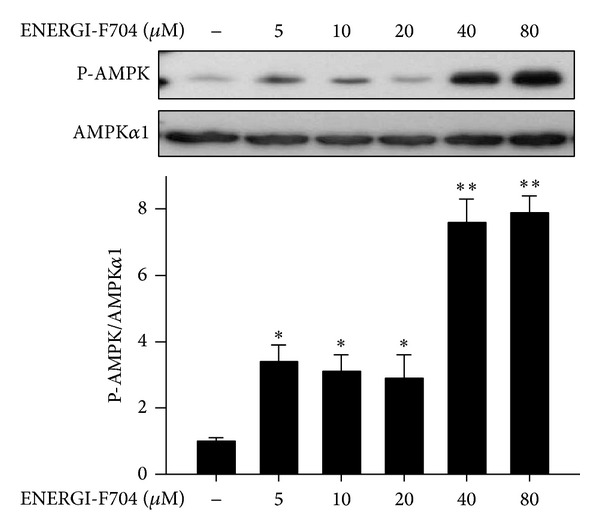
ENERGI-F704 activates AMPK in microglial BV2 cells. BV2 cells were treated with serial concentrations of ENERGI-F704 for 3 h. Cell lysates were used to determine the phosphorylation of AMPK using Western blot analysis. Data are presented as the mean ± SEM of three independent experiments (one-way ANOVA; **, *P* < 0.01; specific comparison to vehicle treated control). Representative images of three independent experiments are shown.

We examined the cytotoxicity of ENERGI-F704 in microglial BV2 cells using XTT assay. As shown in Figure 1, ENERGI-F704 at concentrations up to 80 *μ*M exerted no toxic effect on the BV2 cells treated with or without LPS. We next determined the AMPK activating property of ENERGI-F704 in BV2 cells using Western blot analysis. According to Figure 2, a significant increase in the phosphorylation of AMPK was observed in the presence of ENERGI-F704 in a dose-dependent manner.

### 3.2. ENERGI-F704 Reduces Proinflammatory Cytokines Production in LPS-Induced BV2 Cells

**Figure 3:**
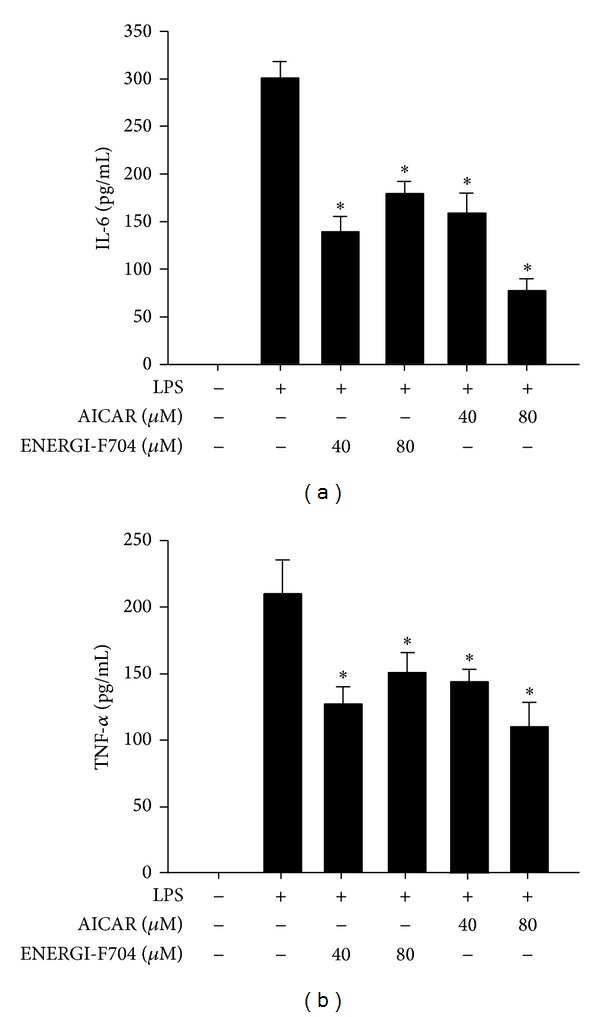
ENERGI-F704 reduced LPS-induced IL-6 and TNF-*α* secretion in BV2 cells. BV2 cells were incubated with 200 ng/mL LPS in the presence of ENERGI-F704 or AICAR for 24 h. The levels of IL-6 (a) and TNF-*α* (b) in culture medium of each condition were accessed using ELISA analysis. Data are presented as the mean ± SEM of three independent experiments (one-way ANOVA; *, *P* < 0.05; specific comparison to LPS-treated control).

AMPK has been reported for its role in suppressing inflammatory responses. Therefore, we next evaluated whether ENERGI-F704 suppresses the production of proinflammatory cytokines such as TNF-*α* and IL-6 in LPS-treated BV2 cells. LPS-treated BV2 cells were treated with either ENERGI-F704 or the pharmacological AMPK activator, 5-aminoimidazole-4-carboxamide 1-*β*-D-ribofuranoside (AICAR), for 24 h. The changes in levels of TNF-*α* and IL-6 were determined using ELISA assay. The exposure of BV-2 cells to LPS resulted in a significant secretion of IL-6 and TNF-*α* after 24 h incubation (Figures 3(a) and 3(b)). The elevations in TNF-*α* and IL-6 production were reduced significantly in the presence of ENERGI-F704. A similar inhibitory phenomenon was observed in the cells treated with AICAR.

### 3.3. ENERGI-F704 Suppresses NO Production and iNOS Expression in LPS-Induced BV2 Cells

**Figure 4:**
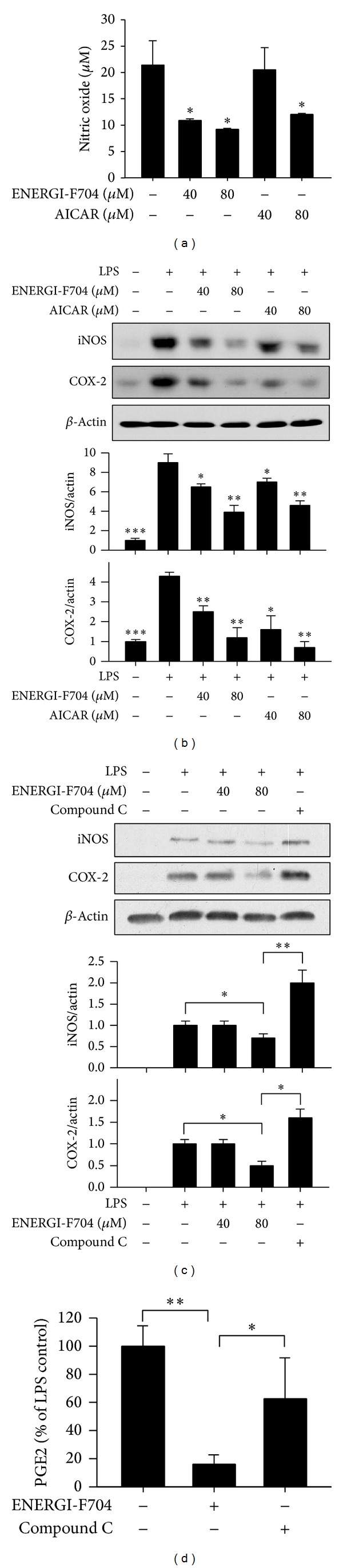
ENERGI-F704 decreases LPS-induced NO, iNOS, COX-2, and PGE2 production in BV2 cells. BV2 cells were stimulated with 200 ng/mL LPS and subsequently treated with either ENERGI-F704 or AICAR for 24 h. (a) Levels of NO in the culture of BV2 cell were determined. (b) After treatments, cell lysates were used to determine the level of iNOS and COX-2 using Western blotting. (c) LPS-treated BV2 cells were treated with ENERGI-F704 in cotreatment with or without compound C for 24 h. Cell lysates were used to determine the level of iNOS and COX-2 using Western blotting. (d) LPS-induced BV2 cells were treated with ENERGI-F704 in cotreatment with or without compound C for 48 h, and the level of PGE2 in the culture medium was determined. Data are presented as the mean ± SEM of three independent experiments (two-way ANOVA; *, **, and ***, *P* < 0.05, *P* < 0.01, and *P* < 0.01; specific comparison to LPS-treated control). Representative images of three independent experiments are shown.

Nitric oxide (NO) acts as signaling molecule in inflammation. The excessive production of NO in response to LPS stimulation might lead to activation of apoptotic signaling in brain tissue [27]. To evaluate the effects of ENERGI-F704 on NO production induced by LPS, BV2 cells stimulated with LPS were treated with ENERGI-F704 or AICAR for 24 h, and concentration of NO was analyzed. As shown in Figure 4(a), treatment with ENERGI-F704 at concentrations of 40 and 80 *μ*M significantly inhibited NO production of LPS-treated BV-2 cells, whereas AICAR inhibited NO production as well. To explore the mechanism underlying inhibitory effect of ENERGI-F704 on LPS-induced NO production, expression of iNOS in each experimental group was assessed using Western blotting. The elevated expressions of iNOS in LPS-induced BV2 cells were significantly reduced by treatment with ENERGI-F704 or AICAR (Figure 4(b)), whereas use of AMPK inhibitor, compound C, led to negatively compensated expression of iNOS (Figure 4(c)).

### 3.4. ENERGI-F704 Inhibited LPS-Induced Cyclooxygenase 2 Expression and PGE2 Production

We further evaluated the effect of ENERGI-F704 on COX-2 expression in BV2 cells challenged with LPS using Western blot assay. As shown in Figure 4(b), the expression of COX-2 was upregulated upon LPS stimulation. The upregulated expression of COX-2 was ameliorated by ENERGI-F704 or AICAR (Figure 4(b)), whereas the treatment with compound C remarkably reversed the suppression (Figure 4(c)). As expected, when measuring the product of COX-2, as shown in Figure 4(d), the concentration of extracellular PGE2 in LPS-induced BV2 cells was diminished by ENERGI-F704, which can be offset in the presence of compound C.

### 3.5. ENERGI-F704 Attenuates LPS-Induced Nuclear Translocation and Production of NF-*κ*B

**Figure 5:**
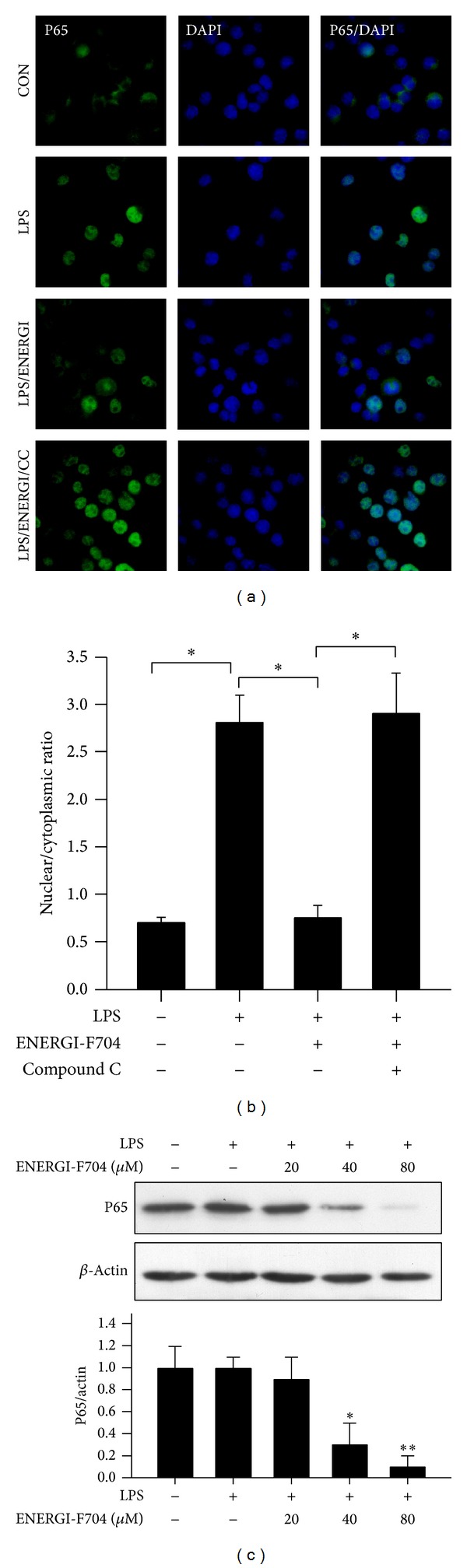
ENERGI-F704 attenuates LPS-induced nuclear translocation and production of NF-*κ*B in BV2 cells. BV2 cells were stimulated with 200 ng/mL LPS and subsequently treated with ENERGI-F704 incorporating with or without compound C for 1 h. (a) After treatments, cells were fixed for immunocytochemical staining. NF-*κ*B and nuclei were visualized using Alexa Fluor 488 (green) and DAPI (blue), respectively. (b) Ratio of nuclear: cytoplasmic immunofluorescence of NF-*κ*B was assessed by microscopy image and quantified using ImageJ software. Ratio < 1 indicates brighter cytoplasmic staining for NF-*κ*B, whereas ratios > 1 indicate brighter nuclear staining for NF-*κ*B. (c) BV2 cells were incubated with 200 ng/mL LPS in the presence of ENERGI-F704 for 24 h. After treatments, cell lysates were used to determine the levels of NF-*κ*B using Western blotting. Data are presented as the mean ± SEM of three independent experiments (one-way ANOVA; * and **, *P* < 0.05 and *P* < 0.01; specific comparison to LPS-treated control). Representative images of three independent experiments are shown.

NF-*κ*B is known for its critical role in inflammation through regulating transcription of proinflammatory cytokines such as IL-1*β*, IL-6, TNF-*α*, and iNOS. To examine the effects of ENERGI-F704 on NF-*κ*B activation, LPS-stimulated BV2 cells were treated with ENERGI-F704 for 1 h and examined for nuclear translocation of NF-*κ*B. As shown in Figure 5, NF-*κ*B stained green retained in cytosol and translocated into nuclei in response to LPS stimulation. The treatment of LPS-induced BV2 cells with ENERGI-F704 attenuated the translocation of NF-*κ*B to nuclei. The inhibitory effect of ENERGI-F704 on NF-*κ*B translocation was reversed in the presence of compound C. Furthermore, our data revealed that the expression level of P65 was reduced significantly in a dose-dependent manner (Figure 5(c)).

**Figure 6:**
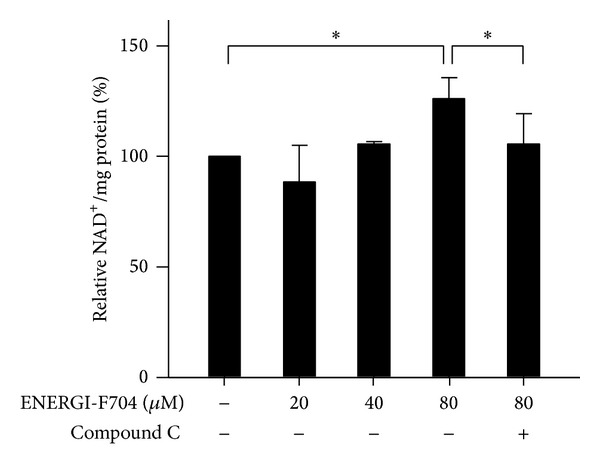
ENERGI-F704 increases the level of NAD in BV2 cells. BV2 cells were incubated with 200 ng/mL LPS in the presence of ENERGI-F704 or compound C for 24 h. Cell lysates collected from each condition were used to determine the level of NAD using NAD/NADPH colorimetric analysis. Data are presented as the mean ± SEM of three independent experiments (one-way ANOVA; *, *P* < 0.05).

Activation of AMPK leads to the deacetylation activity of SIRT1 mediated by the increase of NAD^+^ and acetylation of P65 is required for the stability of NF-*κ*B [28–30]. We next determined the effect of ENERGI-F704 on the NAD^+^ level. As shown in Figure 6, the treatment with ENERGI-F704 dose-dependently increased the level of NAD^+^. Interestingly, increased level of NAD^+^ in response to ENERGI-F704 treatment was largely reversed in the presence of compound C.

## 4. Discussion

Accumulating evidence shows that AMPK is a repressor of inflammation. However, there are limited studies addressed on the effect of AMPK activators on LPS-induced microglia. In the present study, ENERGI-F704, a proprietary compound identified from bamboo shoots extract, exerts AMPK activation activity in human microglial cell model BV2. ENERGI-F704 suppressed LPS-induced IL-6 and TNF-*α* secretion. Moreover, ENERGI-F704 decreased LPS-induced iNOS and COX-2 expression as well as the production of NO and PGE2 in BV2 cells. In the testing condition, our data suggest ENERGI-F704 with anti-inflammatory activity without apparent cytotoxicity. In fact, our previous study has reported that ENERGI-F704 can trigger the phosphorylation of AMPK in human umbilical vein endothelial cells [26]. All the results indicate that ENERGI-F704 acts as an AMPK activator to ameliorate that the NF-*κ*B-involved inflammation response is reliable and not cell type restricted.

Some AMPK activators such as AICAR and metformin have also been demonstrated for their potential to modulate inflammation via NF-*κ*B [25, 31, 32]. Our results had showed ENERGI-F704 as a feasible mean to attenuate LPS-induced inflammatory responses in BV2 and other cell lines. As the anti-inflammatory effects of ENERGI-F704 were diminished in the presence of AMPK inhibitor, compound C, it again suggests that the amelioration of LPS-induced inflammation in microglia BV2 cells by ENERGI-F704 is mediated by AMPK activation. In addition, the activation of AMPK can activate SIRT1 activity via increasing intracellular NAD^+^ levels [28]. It has been demonstrated that SIRT1 could deacetylate p65 subunit of NF-*κ*B complex at lysine 310 and consequently enhance the set-mediated methylation of lysines 314 and 315 [29, 30]. Moreover, the methylation of lysines 314 and 315 resulted in degradation of p65 through triggering ubiquitin-proteasome system. In the present study, we did observe an increase in the level of NAD and a reduction in the level of p65 subunit under the treatment of ENERGI-F704 (Figure 4(d)). Therefore, it is possible that destabilization of p65 protein might be another way of ENERGI-F704 to modulate NF-*κ*B activity. Indeed, the LPS-induced expression of p65 downstream targets, iNOS and COX-2, can be suppressed in the presence of ENERGI-F704 (Figures 4(b) and 4(c)).

Numerous studies showed that manipulation of NF-*κ*B signaling provides beneficial effects in treating neuron injury. It has been examined that suppression of NF-*κ*B activity by using IKK inhibitors, AS602868 and BAY 11-7082, exerts long-lasting protection of primary neurons and oligodendrocytes under N-methyl-D-aspartate induced excitotoxicity [33, 34]. In a mouse model of focal cerebral ischemia, transgenic expression of NF-*κ*B suppressor or pharmacological inhibitor of IKK reduced the infarct size [35]. Besides neuron injury, activation of NF-*κ*B has been found in several aging-related neurodegenerative diseases including Alzheimer's disease and Parkinson's disease, which was considered as a pivotal target for therapy of neurodegenerative diseases [14, 15]. In addition, recent studies further revealed that the neuron immune crosstalk of NF-*κ*B, IKK, and microglia in hypothalamus is important in systemic ageing progression, so immune inhibition can be a potential strategy for lifespan extension [36]. Considering AMPK activation effectively manipulating the NF-*κ*B signaling, AMPK activators, including ENERGI-F704, are potential therapeutic agents for neurodegenerative diseases. Further studies are awaited to elucidate the underlying mechanism of ENERGI-F704 on AMPK.

## 5. Conclusions

In conclusion, ENERGI-F704 suppressed inflammatory responses and induced AMPK activation in LPS-treated BV-2 cells. Anti-inflammatory effect of ENERGI-F704 was attributed to modulating the nuclear translocation and stability of NF-*κ*B. The anti-inflammatory effects of ENERGI-F704 were diminished in the presence of AMPK inhibitor. It is suggested that ENERGI-F704 has a potential to act as anti-inflammatory agent for treating neuroinflammatory diseases through AMPK activation. Further studies are necessary to elucidate the actual mechanism underlying anti-inflammatory activity of ENERGI-F704.
